# Addressing frontline healthcare worker perspectives on hand-hygiene monitoring badges

**DOI:** 10.1017/ash.2023.321

**Published:** 2023-09-29

**Authors:** Tucker Smith, Olivia Hess, Rachel Pryor, Michelle Doll, Gonzalo Bearman

## Abstract

**Background:** Hand-hygiene technology (HHT) intends to monitor and promote hand washing by healthcare workers, a critical measure of infection control. Healthcare worker noncompliance with HHT is a major limitation to its implementation and utility in clinical settings. We assessed perspectives on HHT in an academic hospital system. **Methods:** Hand-hygiene team members created an anonymous, 37-question, Likert-scale survey to assess healthcare worker attitudes toward HHT. Surveys targeted nursing staff, advanced practice providers, care partners, and internal medicine physicians. Clinical coordinators from 5 distinct nursing units and 1 physician department emailed surveys to eligible employees. Research coordinators and clinical coordinators also posted a QR code for survey fliers at nursing stations. **Results:** Overall, 120 surveys were completed. Most surveys were completed by nurses and physicians (66.4% and 14.0%). Most respondents (67.5%) do not find HHT useful. Additionally, 78.3% of respondents believe that HHT does not accurately record hand-washing events. Most (78.3%) do not like using HHT, and 75.8% find it annoying. Only 10.8% believe that patient care suffers because of HHT. **Conclusions:** Most healthcare workers dislike the HHT badges, primarily due to perceived inaccuracies, lack of utility, burden of use, and pressure to comply. Distrust and effect on patient care do not appear to be substantial factors contributing to negative perceptions of HHT. Weaknesses of the study include overrepresentation of nursing staff and potential bias because respondents may have provided exceptionally negative responses believing it could lead to the removal of HHT.

**Figure:**
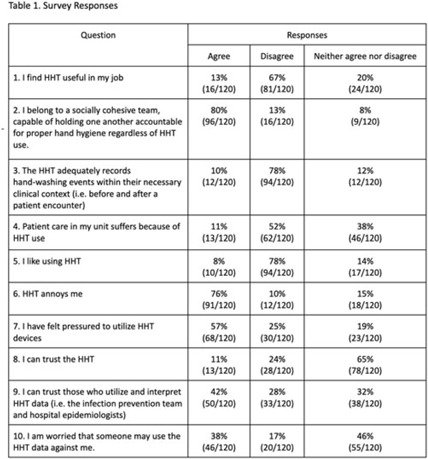

**Disclosures:** None

